# Supplementary material for: On topology and knotty entanglement in protein folding
Source: PLoS One. 2021 Jan 13;16(1):e0244547. doi: 10.1371/journal.pone.0244547 (PMC7806123; doi:10.1371/journal.pone.0244547)
Supplement: S1 File — (PDF) [file pone.0244547.s001.pdf]

S1 File

On topology and knotty entanglement in protein folding

Alexander Begun,[1,*] Sergei Lyubimov,[1,†] Alexander Molochkov,[1,‡] and Antti J. Niemi[2,1,3,§]

[1] *Pacific Quantum Center, Far Eastern Federal University, 690950 Sukhanova 8, Vladivostok, Russia*

[2] *Nordita, Stockholm University, Roslagstullsbacken 23, SE-106 91 Stockholm, Sweden*

[3] *Department of Physics, Beijing Institute of Technology, Haidian District, Beijing 100081, People's Republic of China*

Abstract

We describe in detail how the energy function of the generalized to the nonlinear Schrödinger equation, on which our approach is based, emerges from general considerations.

- Continuum Frenet equation and effect of frame rotations
- Kirchhoff elastic rod and the nonlinear Schrödinger equation
- Topological solitons
- Discrete Frenet equation and the discretized nonlinear Schrödinger equation
- Multi-soliton model of the AFV3-109 protein (used in the article)

[*] beg.alex93@gmail.com

[†] lyubimovsd@gmail.com

[‡] molochkov.alexander@gmail.com

[§] Antti.Niemi@su.se

I. CONTINUUM CURVES AND GENERALIZED KIRCHHOFF'S ELASTIC ROD

A. The Frenet Equation

The geometry of a class $\mathcal{C}^3$ differentiable curve $\mathbf{x}(s)$ in $\mathbb{R}^3$ is governed by the Frenet equation, described widely in elementary courses of differential geometry [1]. We parametrize the curve with its proper length $s \in [0, L]$ where L is the length of the curve in $\mathbb{R}^3$. We introduce the unit length tangent vector

$$\mathbf{t} \;=\; \frac{d\mathbf{x}(s)}{ds} \;\equiv\; \mathbf{x}_s \tag{1}$$

the unit length bi-normal vector

$$\mathbf{b} \;=\; \frac{\mathbf{x}_s \times \mathbf{x}_{ss}}{||\mathbf{x}_s \times \mathbf{x}_{ss}||} \tag{2}$$

and the unit length normal vector,

$$\mathbf{n} = \mathbf{b} \times \mathbf{t} \tag{3}$$

The three vectors $(\mathbf{n}, \mathbf{b}, \mathbf{t})$ define the orthonormal, right-handed Frenet frames. We can introduce this framing at every point along the curve, whenever

$$\mathbf{x}_s \times \mathbf{x}_{ss} \;\neq\; 0 \tag{4}$$

The Frenet equation transports the frames along the curve as follows,

$$\frac{d}{ds}\begin{pmatrix} \mathbf{n} \\ \mathbf{b} \\ \mathbf{t} \end{pmatrix} \;=\; \begin{pmatrix} 0 & \tau & -\kappa \\ -\tau & 0 & 0 \\ \kappa & 0 & 0 \end{pmatrix} \begin{pmatrix} \mathbf{n} \\ \mathbf{b} \\ \mathbf{t} \end{pmatrix} \tag{5}$$

Here

$$\kappa(s) \;=\; \frac{||\mathbf{x}_s \times \mathbf{x}_{ss}||}{||\mathbf{x}_s||^3} \tag{6}$$

is the curvature and

$$\tau(s) \;=\; \frac{(\mathbf{x}_s \times \mathbf{x}_{ss}) \cdot \mathbf{x}_{sss}}{||\mathbf{x}_s \times \mathbf{x}_{ss}||^2} \tag{7}$$

is the torsion. Both $\kappa(s)$ and $\tau(s)$ are extrinsic geometric quantities *i.e.* they depend only on the shape of the curve in $\mathbb{R}^3$. Conversely, if we know the curvature and torsion we can construct the curve, by first solving for $\mathbf{t}(s)$ from the Frenet equation followed by integration of (1). The solution is unique, modulo a global translation and rotation.

B. Frame rotation

We start with the observation that the normal and bi-normal vectors do not appear in (1). As a consequence a rotation around $\mathbf{t}(s)$,

$$\begin{pmatrix} \mathbf{n} \\ \mathbf{b} \end{pmatrix} \rightarrow \begin{pmatrix} \mathbf{e}_1 \\ \mathbf{e}_2 \end{pmatrix} = \begin{pmatrix} \cos\eta(s) & \sin\eta(s) \\ -\sin\eta(s) & \cos\eta(s) \end{pmatrix} \begin{pmatrix} \mathbf{n} \\ \mathbf{b} \end{pmatrix}. \tag{8}$$

has no effect on the curve. For the Frenet equation this rotation gives

$$\frac{d}{ds}\begin{pmatrix} \mathbf{e}_1 \\ \mathbf{e}_2 \\ \mathbf{t} \end{pmatrix} = \begin{pmatrix} 0 & (\tau+\partial_s\eta) & -\kappa\cos\eta \\ -(\tau+\partial_s\eta) & 0 & -\kappa\sin\eta \\ \kappa\cos\eta & \kappa\sin\eta & 0 \end{pmatrix} \begin{pmatrix} \mathbf{e}_1 \\ \mathbf{e}_2 \\ \mathbf{t} \end{pmatrix}. \tag{9}$$

The form of (9) suggests to combine the two κ dependent contributions into a single complex quantity [2–4],

$$\kappa \xrightarrow{\ \eta\ } \kappa(\cos\eta + i\sin\eta) \equiv \kappa e^{i\eta} \tag{10}$$

We may then introduce the following notations/conventions when representing curvature and torsion in arbitrary frame,

$$\begin{aligned} \kappa \rightarrow \kappa e^{-i\eta} &\equiv \phi \\ \tau \rightarrow \tau + \partial_s\eta &\equiv \sqrt{\tfrac{d}{2}}A_i \end{aligned} \tag{11}$$

Here d is a parameter that we introduce for future convenience; for the Frenet equations we may set $d = 2$. With these variables, (9) admits the manifestly frame covariant form:

$$\begin{aligned} (\tfrac{d}{ds} \mp i\sqrt{\tfrac{d}{2}}A)\mathbf{e}_\pm &= -\phi\mathbf{t} \\ \tfrac{d}{ds}\mathbf{t} &= \tfrac{1}{2}(\phi\,\mathbf{e}_+ + \bar{\phi}\,\mathbf{e}_-) \end{aligned} \tag{12}$$

with

$$\mathbf{e}_\pm = \mathbf{e}_1 \pm i\mathbf{e}_2 \qquad \Rightarrow \qquad \mathbf{e}_\pm \rightarrow e^{\pm i\eta}\mathbf{e}_\pm$$

and we remind that $\mathbf{t}$ is frame invariant.

C. The Kirchhoff elastic rod and its generalizations

The curvature and torsion are the only quantities available to construct energy functions for filamentous, inextensible elastic rods. According to Kirchhoff the energy is [5]

$$E = \int_0^L ds\{\alpha\kappa^2 + \beta\tau^2\} \tag{13}$$

where α and β are some parameters. The case $\beta = 0$ corresponds to Euler's elastica; in a biological context this defines the worm like chain (WLC) model that is commonly used to describe long and flexible linear (bio)polymers [2]

The energy function (13) describes the bending and twisting of a thin rod in the limit of very small curvature and torsion. But this energy function is not capable of describing phenomena such a supercoiling, nor structures such as helix-loop-helix that are common in case of proteins. For this we need to include higher order, non-linear contributions to (13). To do this systematically, we need a guiding principle: Note that even though framing is a necessary intermediate step to construct the curve from the knowledge of its curvature and torsion, the shape of a curve can not depend on the way how it is framed. Indeed, the Frenet equations can be presented in the frame covariant form (12). Thus, the energy function should similarly admit a frame covariant form, one that is the same independently of the framing when expressed in the frame covariant variables (ϕ, A) in (11). An example of a frame covariant energy function is [2–4],

$$H = \int_0^L ds \left\{ |(\partial_s + i\sqrt{\frac{d}{2}}A)\phi|^2 + \lambda\,(|\phi|^2 - m^2)^2 \; - \; a\,A + \frac{c}{2}A^2 \right\} \tag{14}$$

The first two terms have the functional form of the Hamiltonian that appears in the Abelian Higgs model. They remain *manifestly* intact under a frame rotation (11).

The third term, with parameter a, is the one dimensional Chern-Simons term. It breaks chirality which ensures that the curves are chiral, either right-handed or left-handed depending on the sign of parameter a. Note that under a frame rotation this terms transforms by a derivative; see (11). Thus it remains invariant when there are no end point frame rotations.

The fourth term in (14) is called the Proca mass in the context of the Abelian Higgs model. It is *not* covariant under a frame rotation but we included it for completeness since it yields the second term in (13), in Frenet frames.

D. Energy and soliton of Nonlinear Schrödinger equation

In term of the geometric curvature and torsion, the energy density of (14) translates to

$$\mathcal{H} \; = \; (\partial_s \kappa)^2 + \frac{d}{2}\kappa^2\tau^2 + \lambda\,(\kappa^2 - m^2)^2 \; - \; a\,\tau \; + \; \frac{c}{2}\,\tau^2 \tag{15}$$

We introduce the Hasimoto variable [3, 4, 6], to combine the curvature and torsion into a single frame invariant complex quantity

$$\psi(s) \; = \; \kappa(s)\exp\{i\int\limits_0^s ds'\,\tau(s')\} \; \equiv \; \phi(s)\exp\{i\sqrt{\frac{d}{2}}\int\limits_0^s ds'\,A(s')\} \tag{16}$$

In terms of (16), we find that (15) includes the following,

$$(\partial_s \kappa)^2 + e^2\kappa^2\tau^2 + \lambda\kappa^4 \; = \; \bar\psi_s\psi_s + \lambda(\bar\psi\psi)^2 \; = \; \mathcal{H}_3 \tag{17}$$

This the energy density of the standard nonlinear Schrödinger equation (NLS), the paradigm integrable model that supports solitons as classical solutions: The non-vanishing Poisson bracket of the Hasimoto variables is

$$\{\psi(s), \bar\psi(s')\} \; = \; i\delta(s - s')$$

and the following quantities are conserved densities in the sense that their Poisson brackets with $\mathcal{H}_3$ vanish [3, 4, 6]

$$\begin{aligned} \mathcal{H}_{-2} &= \tau \\ \mathcal{H}_{-1} &= L \\ \mathcal{H}_1 &= \kappa^2 \quad\;\; \sim \bar\psi\psi \\ \mathcal{H}_2 &= i\kappa^2\tau \;\;\, \sim \bar\psi\psi_s \end{aligned} \tag{18}$$

The energy (15) is a combination of $\mathcal{H}_{-2}$, $\mathcal{H}_1$ and $\mathcal{H}_3$, except for its last term, the Proca mass. From the perspective of the NLS hierarchy, the momentum $\mathcal{H}_2$ should also be included so that at the end we have the energy density

$$\mathcal{H} \; = \; (\partial_s \kappa)^2 + \frac{d}{2}\kappa^2\tau^2 + \lambda\,(\kappa^2 - m^2)^2 \; - \; b\kappa^2\tau \; - \; a\tau \; + \; \frac{c}{2}\tau^2 \tag{19}$$

The standard NLS equation is the paradigm equation that supports solitons [7, 8]; depending on the sign of λ the soliton is either dark ($\lambda > 0$) or bright ($\lambda < 0$). In particular, the torsion independent contribution

$$(\partial_s \kappa)^2 + \lambda\,(\kappa^2 - m^2)^2 \tag{20}$$

supports the double well *topological* soliton: When m^2 is positive and when κ can take both positive and negative values, the equation of motion

$$\partial_{ss}\kappa = 2\lambda\kappa(\kappa^2 - m^2)$$

is solved by

$$\kappa(s) \;=\; m\,\tanh\left[m\sqrt{\lambda}(s-s_0)\right] \tag{21}$$

The energy function (19) is quadratic in the torsion. Thus we can eliminate τ using its equation of motion,

$$\tau[\kappa] \;=\; \frac{a+b\kappa^2}{c+d\kappa^2} \;\equiv\; \frac{a}{c}\,\frac{1+(b/a)\kappa^2}{1+(d/c)\kappa^2} \tag{22}$$

and we obtain the following equation of motion for curvature,

$$\kappa_{ss} = V_\kappa[\kappa] \tag{23}$$

where

$$V[\kappa] \;=\; -\left(\frac{bc-ad}{d}\right)\frac{1}{c+d\kappa^2} \;-\; \left(\frac{b^2+8\lambda m^2}{2b}\right)\kappa^2 + \lambda\,\kappa^4 \tag{24}$$

This shares the same large-κ asymptotics, with the potential in (20). With properly chosen parameters, we expect that (23), (24) continue to support topological solitons, but we do not know their explicit profile, in terms of elementary functions.

The curve is the constructed as follows: Once we have the soliton of (23), we evaluate $\tau(s)$ from (22). We substitute the ensuing (κ,τ) profiles in the Frenet equation (5) and solve for $\mathbf{t}(s)$. We then integrate (1) to obtain the curve $\mathbf{x}(s)$ that corresponds to the soliton. A generic soliton curve looks like a helix-loop-helix motif (more generally a regular secondary structure - a loop - a regular secondary structure), familiar from crystallographic protein structures.

II. POLYGONS AND GENERALIZED KIRCHHOFF ENERGIES

A. Discrete Frenet equation

Proteins are not alike continuous, differentiable curves. Proteins are like piecewise linear polygonal chain. Thus, to construct a generalized Kirchhoff model applicable for proteins, we need to generalise the Frenet frame formalism to the case of a polygonal, piecewise linear chain [9].

Let $\mathbf{r}_i$ with $i = 1, ..., N$ be the vertices of the chain. At each vertex we introduce the unit tangent vector

$$\mathbf{t}_i = \frac{\mathbf{r}_{i+1} - \mathbf{r}_i}{|\mathbf{r}_{i+1} - \mathbf{r}_i|} \tag{25}$$

the unit binormal vector

$$\mathbf{b}_i = \frac{\mathbf{t}_{i-1} - \mathbf{t}_i}{|\mathbf{t}_{i-1} - \mathbf{t}_i|} \tag{26}$$

and the unit normal vector

$$\mathbf{n}_i = \mathbf{b}_i \times \mathbf{t}_i \tag{27}$$

The orthonormal triplet $(\mathbf{n}_i, \mathbf{b}_i, \mathbf{t}_i)$ defines a discrete version of the Frenet frames (1)-(3) at each position $\mathbf{r}_i$ along the chain.

In lieu of the curvature and torsion, we have their discrete analogues, the bond angles and torsion angles. When we know the vertices we also know the Frenet frames and we can compute these angles: The bond angles are

$$\theta_i \equiv \theta_{i+1,i} = \arccos(\mathbf{t}_{i+1} \cdot \mathbf{t}_i) \tag{28}$$

and the torsion angles are

$$\phi_i \equiv \phi_{i+1,i} = \text{sign}\{\mathbf{b}_{i-1} \times \mathbf{b}_i \cdot \mathbf{t}_i\} \cdot \arccos(\mathbf{b}_{i+1} \cdot \mathbf{b}_i) \tag{29}$$

Conversely, when the values of the bond and torsion angles are all known, we can use the discrete version of the Frenet equation (5)

$$\begin{pmatrix} \mathbf{n}_{i+1} \\ \mathbf{b}_{i+1} \\ \mathbf{t}_{i+1} \end{pmatrix} = \begin{pmatrix} \cos\theta\cos\phi & \cos\theta\sin\phi & -\sin\theta \\ -\sin\phi & \cos\phi & 0 \\ \sin\theta\cos\phi & \sin\theta\sin\phi & \cos\theta \end{pmatrix}_{i+1,i} \begin{pmatrix} \mathbf{n}_i \\ \mathbf{b}_i \\ \mathbf{t}_i \end{pmatrix} \tag{30}$$

to compute the frame at position $i + i$ from the frame at position i. Once all the frames have been constructed, the entire string is given by discrete version of (1),

$$\mathbf{r}_k = \sum_{i=0}^{k-1} |\mathbf{r}_{i+1} - \mathbf{r}_i| \cdot \mathbf{t}_i \tag{31}$$

In the case of a protein, it is sufficient to take $|\mathbf{r}_{i+1} - \mathbf{r}_i| = 3.8\text{Å}$; this is the average distance between neighboring Cα atoms. The bond oscillations are very fast, and over time intervals in the scale of microsecond the average values can be used.

In constructing the chain, without any loss of generality we may choose $\mathbf{r}_0 = 0$, make $\mathbf{t}_0$ to point into the direction of the positive z-axis, and let $\mathbf{t}_1$ lie on the y-z plane.

B. frame rotations

The vectors $\mathbf{n}_i$ and $\mathbf{b}_i$ do not appear in (31). Thus, as in the case of continuum curves, a discrete chain remains intact under frame rotations of the $(\mathbf{n}_i, \mathbf{b}_i)$ zweibein around $\mathbf{t}_i$. This local SO(2) rotation acts on the frames as follows [9]

$$\begin{pmatrix} \mathbf{n} \\ \mathbf{b} \\ \mathbf{t} \end{pmatrix}_i \to e^{\Delta_i T^3} \begin{pmatrix} \mathbf{n} \\ \mathbf{b} \\ \mathbf{t} \end{pmatrix}_i = \begin{pmatrix} \cos\Delta_i & \sin\Delta_i & 0 \\ -\sin\Delta_i & \cos\Delta_i & 0 \\ 0 & 0 & 1 \end{pmatrix} \begin{pmatrix} \mathbf{n} \\ \mathbf{b} \\ \mathbf{t} \end{pmatrix}_i \tag{32}$$

Here Δ_i is the rotation angle at vertex i and T^3 is one of the SO(3) generators

$$T^1 = \begin{pmatrix} 0 & 0 & 0 \\ 0 & 0 & -1 \\ 0 & 1 & 0 \end{pmatrix} \quad T^2 = \begin{pmatrix} 0 & 0 & 1 \\ 0 & 0 & 0 \\ -1 & 0 & 0 \end{pmatrix} \quad T^3 = \begin{pmatrix} 0 & -1 & 0 \\ 1 & 0 & 0 \\ 0 & 0 & 0 \end{pmatrix}$$

that satisfy the Lie algebra

$$[T^a, T^b] = \epsilon^{abc} T^c$$

Using these matrices we can write the effect of frame rotation on the bond and torsion angles as follows

$$\theta_i \, T^2 \ \to \ e^{\Delta_i T^3} (\theta_i T^2) \, e^{-\Delta_i T^3} \tag{33}$$

$$\phi_i \ \to \ \phi_i + \Delta_{i-1} - \Delta_i \tag{34}$$

Since the $\mathbf{t}_i$ remain intact under (32), the gauge transformation of (θ_i, ϕ_i) has no effect on the geometry of the discrete string.

A priori, the fundamental range of the bond angle is $\theta_i \in [0, \pi]$ while for the torsion angle the range is $\phi_i \in [-\pi, \pi)$. Thus we identify (θ_i, ϕ_i) as the canonical latitude and longitude angles of a two-sphere $\mathbb{S}^2$. For practical purposes we find it useful to extend the range of θ_i into negative values $\theta_i \in [-\pi, \pi]\ mod(2\pi)$. We compensate for this two-fold covering of $\mathbb{S}^2$ by a $\mathbb{Z}_2$ symmetry which takes the following form:

$$\begin{aligned} \theta_k \ &\to \ -\theta_k \qquad \text{for all} \ \ k \geq i \\ \phi_i \ &\to \ \phi_i - \pi \end{aligned} \tag{35}$$

This is a special case of (33), (34), with

$$\begin{aligned} \Delta_k &= \pi \qquad \text{for} \ \ k \geq i+1 \\ \Delta_k &= 0 \qquad \text{for} \ \ k < i+1 \end{aligned}$$

C. Generalized discrete Kirchhoff energy and solitons

The energy function used in the article is obtained by a direct naive discretization of (19), and by replacing curvature and torsion by the discrete bond and torsion angles [2, 4, 9] . In particular, we use

$$(\partial_s \kappa)^2 \;\to\; (\theta_{i+1} - \theta_i)^2$$

Thus,

$$(\partial_s \kappa)^2 + \lambda\,(\kappa^2 - m^2)^2 \;+\; \frac{d}{2}\kappa^2\tau^2 \;-\; b\kappa^2\tau \;-\; a\tau \;+\; \frac{c}{2}\tau^2$$

becomes

$$\sum_{k=1}^{k_{max}} \left\{ \sum_{i=1}^{n} \left(-2\theta_{i+1}\theta_i + 2\theta_i^2 + \lambda_k\,(\theta_i^2 - m_k^2)^2 + \frac{d_k}{2}\,\theta_i^2\phi_i^2 - b_k\,\theta_i^2\phi_i - a_k\,\phi_i + \frac{c_k}{2}\phi_i^2 \right) \right\} \tag{36}$$

which is the (θ, ϕ) contribution to the energy function in Eqn. (4) of the article. Note that as explained in the article, we have added here a summation over k, to account for the fact that in the case of proteins we have a chain that is made of k_{max} consecutive segments, each with its own set of parameters. Normally, these segments correspond to the different super-secondary helix-loop-helix, strand-loop-strand *etc* motifs, in the case of a protein.

The conventional discrete NLS equation is known to support solitons [10]. Thus we expect that (36) supports soliton solutions as well: We follow (22) to eliminate the torsion angle (we suppress the index k)

$$\phi_i[\theta] \;=\; \frac{a + b\theta_i^2}{c + d\theta_i^2} \;=\; a\frac{1 + (b/a)\theta_i^2}{c + d\theta_i^2} \tag{37}$$

For bond angles we then have

$$\theta_{i+1} = 2\theta_i - \theta_{i-1} + \frac{dV[\theta]}{d\theta_i^2}\theta_i \qquad (i = 1, ..., N) \tag{38}$$

We set $\theta_0 = \theta_{N+1} = 0$, and $V[\theta]$ is given by (24). To solve this numerically, we use the iterative equation [2, 11]

$$\theta_i^{(n+1)} = \theta_i^{(n)} - \epsilon \left\{ \theta_i^{(n)} V'[\theta_i^{(n)}] - (\theta_{i+1}^{(n)} - 2\theta_i^{(n)} + \theta_{i-1}^{(n)}) \right\} \tag{39}$$

where $\{\theta_i^{(n)}\}_{i\in N}$ is the nth iteration of an initial configuration $\{\theta_i^{(0)}\}_{i\in N}$ and ϵ is some sufficiently small but otherwise arbitrary numerical constant. We choose $\epsilon = 0.01$, in our simulations. The fixed point of (39) is independent of the value of ϵ, and clearly a solution of (38).

Once the fixed point is found, the corresponding torsion angles are obtained from (37). The frames are then constructed from (30), and the entire chain is constructed using (31).

We do not know of an analytical expression of the soliton solution to the equation (38). But an *excellent* approximative solution can be obtained by discretizing the topological soliton (21) [2]:

$$\theta_i \approx \frac{\mu_1 \cdot e^{\gamma_1(i-s)} - \mu_2 \cdot e^{-\gamma_2(i-s)}}{e^{\gamma_1(i-s)} + e^{-\gamma_2(i-s)}} \tag{40}$$

Here $(\gamma_1, \gamma_2, \mu_1, \mu_2, s)$ are parameters. The μ_1 and μ_2 specify the asymptotic θ_i-values of the soliton. Thus, these parameters are entirely determined by the character of the regular, constant bond and torsion angle structures that are adjacent to the soliton. In particular, these parameters are not specific to the soliton *per se*, but to the adjoining regular structures. The parameter s defines the location of the soliton along the string. This leaves us with only two loop specific parameter, the γ_1 and γ_2. These parameters quantify the length of the bond angle profile that describes the soliton.

For the torsion angle, (37) involves one parameter (a) that we have factored out as the overall relative scale between the bond angle and torsion angle contributions to the energy. This parameter determines the relative flexibility of the torsion angles, with respect to the bond angles. Then, there are three additional parameters $(b/a, c/a, d/a)$ in the remainder $\phi[\theta]$. Two of these are again determined by the character of the regular structures that are adjacent to the soliton. As such, these parameters are not specific to the soliton. The remaining single parameter specifies the size of the regime where the torsion angle fluctuates.

On the regions adjacent to a soliton, we have constant values of (θ_i, ϕ_i). In the case of a protein, these are the regions that correspond to the standard regular secondary structures. For example, the standard right-handed α-helix is obtained by setting

$$\alpha - \text{helix}: \quad \begin{cases} \theta \approx \frac{\pi}{2} \\ \phi \approx 1 \end{cases} \tag{41}$$

and for the standard β-strand

$$\beta - \text{strand}: \quad \begin{cases} \theta \approx 1 \\ \phi \approx \pi \end{cases} \tag{42}$$

All the other standard regular secondary structures of proteins such as 3/10 helices, left-handed helices *etc.* are similarly modeled by definite constant values of θ_i and ϕ_i. Protein loops correspond to solitons, the regions where the values of (θ_i, ϕ_i) are variable.

The presence of solitons *significantly* reduces the number of parameters in (36), increasing the predictive power. In particular, the number of parameters is usually far smaller than the number of amino acids, along the protein backbone.

III. MULTI-SOLITON AND THE FV3-109 BACKBONE

To construct the multi-soliton solution of (38), (37) that models the Cα backbone of a given crystallographic structure, here the slipknotted 2J6B, we start by identifying the individual solitons. We then combine the individual solitons into a single multi-soliton solution of the pertinent DNLS equation. For this we use a combination of the `GaugeIT` and `Propro` packages, described at

$$\texttt{https}://\texttt{protoin.ru/propro/index.php}$$

We start the analysis with an inspection of the bond and torsion angle spectrum, to identify the individual solitons. For this we use the $\mathbb{Z}_2$ symmetry (35), that we implement with the `GaugeIT` package. In the ideal case, each super-secondary structure such as a helix-loop-helix, strand-loop-strand, helix-loop-strand *etc* motif is a single soliton; an ideal length single soliton loop seems to have six residues. Thus, the number of these motifs gives a lower bound to the number of solitons: Since the N and C terminals are generically unstructured the number of solitons must exceed the number of regular helix, strand *etc.* structures, at least by one. In the case of 2J6B this means we need to have at least nine solitons.

But in a given protein structure, a loop can also be very long, it can extend over several Cα atoms; in the case of 2J6B the longest loop extends over sixteen Cα atoms, starting at residue 58 and ending at residue 73. A single long loop may be a combination of several individual solitons. Regular structures such as α-helices and β-strands can also have bends in their middle, so that the values of bond and torsion angles along them are not constant but deviate from the ideal values (41) and (42), for some residues. Depending on the situation, one may then interpret a localized bend along a helix or strand as a soliton, albeit a "shallow" one. Thus there may be more solitons along the backbone, than what is suggested by a *naive* counting of the regular helices, strands *etc.* This number can be estimated by an inspection of the bond and torsion angles, how their profiles react to the $\mathbb{Z}_2$ symmetry transformation (35): Over a soliton profile the bond and torsion angles are always variable.

To estimate an upper bound to the number of solitons, we start with the following observation: Since each soliton must carry at least one *fully independent* pair of bond and torsion angles and it takes four Cα atoms to define a fully independent pair of (θ_i, ϕ_i), the number of solitons is at most as large as the number of Cα atoms divided by four. In particular, the minimum length of a soliton is four Cα atoms. The actual upper bound is then determined by the accuracy at which the experimental structure is measured. For this, a good criterion that we also use in the article is the following. The RMS distance between a crystallographic structure and its multi-soliton description should not be much smaller than the resolution at which the crystallographic structure has been measured. Simply because a model should not be better than the object it describes. In the case of 2J6B, the structure is measured with a 1.30 Å resolution. Thus we increase the number of individual solitons until we obtain a multi-soliton that describes the 2J6B Cα backbone with a comparable RMS precision.

Note that this also means, the multi-soliton representation of a protein backbone is not unique: Two different multi-soliton representations that are both within the experimental resolution from a given crystallographic structure, are both acceptable.

In Figure 1 we show the (θ_i, π_i) spectrum both for 2J6B, and for the multi-soliton we have used in the article. In presenting each, we have used the $\mathbb{Z}_2$ symmetry transformation to convert the bond angles to positive values. There are 20 individual solitons in the multi-soliton, and the Cα RMS distance between the crystallographic structure and the multi-soliton we have constructed using the *Propro* package is around 1.23 Å. This is a very good match to the resolution in the crystallographic structure. One could probably optimize the multi-soliton structure further, and still have RMSD that is compatible with the 1.30 Å resolution of the crystallographic structure. But here our aim is not to find an *optimal* multi-soliton, only one that functions for our purposes. As we aim to simulate the folding of a structure where we know that *e.g.* Gõ models have problems, it should be important to us to construct a multi-soliton with very high precision.

In Table I we show the parameter values that we have used. Note that in this Table, there are two values λ_1 and λ_2, and two values m_1 and m_2 for each soliton. This is because the solitons are asymmetric, with respect to their center: In the approximative solution (40) we have different values μ_1, μ_2 and γ_1, γ_2 that determine the profile of θ_i for values of i that are smaller or larger than s which defines the center of the structure. A comparison with

(21) shows that these two parameters depend on the values of λ and m in (19). Thus, to account for the asymmetry of a loop structure, we also introduce two pairs (λ_1, m_1) and (λ_2, m_2). The first pair describes the soliton from its start to the center, the second pair covers the rest of the soliton.

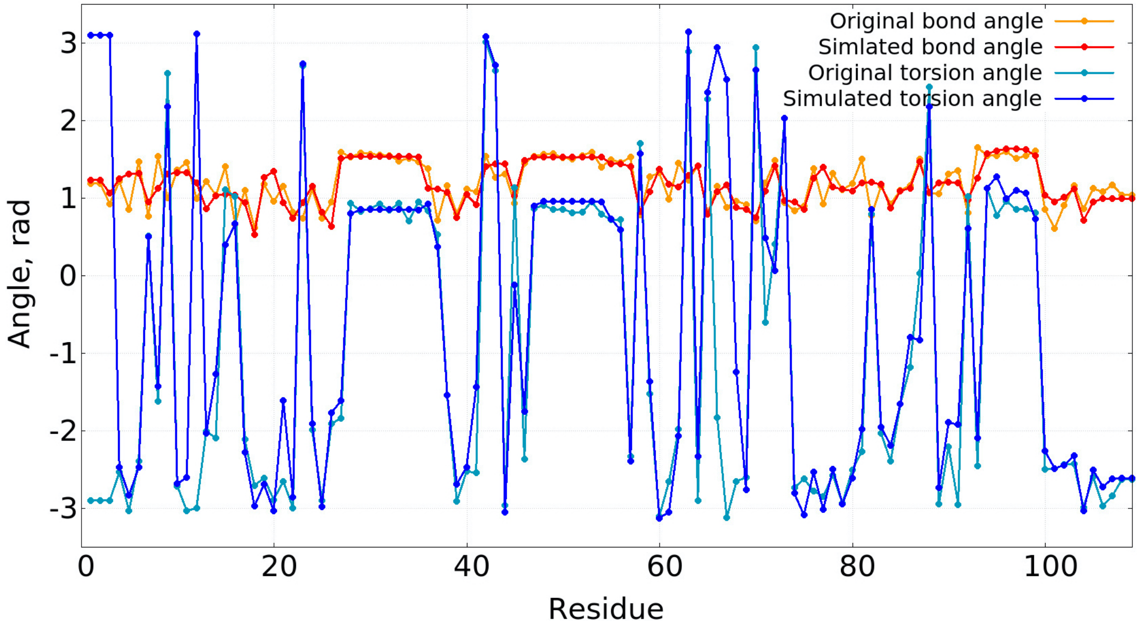

FIG. 1: *Color online:* The bond (θ) and torsion (ϕ) angle spectrum of the PDB structure 2J6B together with the corresponding spectrum of the multi-soliton. Note that the angles are defined modulo 2π.

The evaluation of the parameter values that corresponds to the construction of the multi-soliton proceeds with the *Propro* package, for a given profile that we have identified with *GaugeIT*.

Finally, we comment on the various versions of the Gõ model [13]. These approaches have played a very important rôle, to gain insight to protein folding in particular when the power of computers is insufficient for any kind of serious all-atom folding simulations. In these models the individual atomic coordinates of the folded protein chain commonly appear as an input. A simple energy function is then introduced, tailored to ensure that the known folded configuration is a minimum energy ground state; the energy could be as simple as a square well potential which is centered at the atomic coordinates of the native

number	start	center	end	d/2	λ_1	λ_2	a	c/2	b	m_1	m_2
1	1	3	5	1.5187e-09	2.4025	5.4724	-6.6922e-09	2.9489e-12	1.4833e-07	1.2397	1.3101
2	6	7	9	7.9776e-10	0.9258	1.7811	-2.5802e-08	5.9114e-12	9.1620e-08	1.3671	1.3143
3	10	12	15	3.4764e-10	2.3854	3.1773	-5.6462e-09	7.7903e-12	3.6777e-08	1.3288	1.0405
4	16	17	20	2.9677e-10	2.9205	0.3081	-1.4328e-08	1.9208e-11	4.5343e-08	1.0452	1.5549
5	21	22	24	9.5052e-10	3.1654	1.1811	-3.0383e-09	6.1927e-12	8.3254e-08	0.9222	1.2361
6	25	26	35	6.6137e-10	6.3999	8.0030	-5.3036e-09	3.8382e-11	6.3016e-08	0.8009	1.5335
7	36	38	40	7.0689e-11	9.4063	1.5612	-7.4524e-09	2.1676e-11	1.3793e-08	1.1125	1.1002
8	41	41	44	2.1606e-10	5.0945	7.1494	-1.7556e-08	4.9406e-11	3.1164e-08	1.0318	1.4407
9	45	45	54	4.4705e-10	0.5580	6.9161	-3.0270e-09	1.3689e-11	4.2011e-08	1.6991	1.5232
10	55	57	59	3.5138e-11	7.5205	2.5381	-2.6297e-08	7.1839e-11	1.8906e-08	1.4394	1.0697
11	60	61	63	5.9600e-10	1.4666	2.6355	-1.6514e-09	8.5212e-13	5.7346e-08	1.4128	1.2893
12	64	65	67	1.1694e-09	0.3841	3.5258	-1.2913e-08	5.0953e-12	1.0472e-07	1.6354	1.1806
13	68	70	72	1.3131e-09	6.5289	0.5200	-1.0253e-08	3.6908e-12	1.3901e-07	0.8574	1.5837
14	73	75	77	6.3872e-14	6.5792	1.6986	-3.0860e-10	1.9025e-11	2.3176e-09	0.9493	1.4216
15	78	79	81	5.2001e-11	17.137	3.7579	-1.4679e-09	6.0883e-11	1.0657e-08	1.1341	1.2000
16	82	83	86	5.0164e-12	12.456	2.3005	-3.7847e-08	4.1765e-10	8.2773e-08	1.1998	1.1003
17	87	87	91	8.5997e-11	3.6053	3.8840	-1.1426e-09	9.3701e-12	8.5883e-09	1.5506	1.1987
18	92	92	95	9.9176e-10	1.0386	0.7653	-2.6735e-09	2.2889e-12	9.0914e-08	1.3914	1.6042
19	96	100	102	5.9589e-10	0.4436	9.5105	-1.6451e-08	1.5778e-11	6.0421e-08	1.6378	1.0034
20	103	104	109	1.2532e-09	0.8811	9.7042	-5.5504e-09	1.0922e-12	1.0943e-07	1.2245	0.9898

TABLE I: The parameters in the energy function for 2J6B

conformation. Since the positions of all the relevant atoms appear as parameters in these models, they contain more parameters than unknown and thus no predictions can be made; in the case of 2J6B there are typically around 200 parameters. Only a description is possible. From the point of view of a system of equations, these models are over-determined. In any *predictive* energy function the number of adjustable parameters must remain *smaller* than the number of independent atomic coordinates.

---

[1] M. Spivak, *A Comprehensive Introduction to Differential Geometry* (Five Volumes) 3rd ed. (Publish or Perish, Inc. Berkeley, CA, U.S.A., 1999)

[2] A.J. Niemi, in C. Chamon, M.O. Goerbig, R. Moessner, L.F. Cugliandolo (Eds.) *Topological Aspects of Condensed Matter Physics: Lecture Notes of the Les Houches Summer School* Vol. 103 (Oxford University Press, Oxford, 2017)

[3] Hu, S., Jiang, Y. & Niemi, A.J., *Phys. Rev.* **D87** 105011 (2013)

[4] Ioannidou, T., Jiang, Y., & Niemi, A.J., *Phys. Rev.* **D90** 025012 (2014)

[5] Dill, E.H., *Arch. Hist. Ex. Sci.* **44** 1(1992)

[6] Langer, J., Singer, D., *SIAM Rev.* **38** 605 (1996)

[7] L.A. Takhtadzhyan, L.D. Faddeev, *Hamiltonian approach to soliton theory* (Springer Verlag, Berlin, 1987)

[8] N. Manton and P. Sutcliffe, *Topological Solitons* (Cam- bridge University Press, Cambridge, 2004)

[9] Hu, S., Lundgren, M. & Niemi, A.J., *Phys. Rev.* **E83** 061908 (2011)

[10] P.G. Kevrekidis, *The discrete Nonlinear Schödinger equation: Mathematical Analysis, Numerical Computations and Physical Perspectives* (Springer Verlag, Berlin, 2009)

[11] Molkenthin, N., Hu, S. & Niemi, A.J., *Phys. Rev. Lett.* **106** 078102 (2011)

[12] Peng, X., Sieradzan, A. & Niemi, A.J., *Phys. Rev* **E94** 062405 (2016)

[13] Gō, N. N., *Annual Review of Biophysics and Bioengineering* **12** 183(1983)
