# Supplementary material for: On topology and knotty entanglement in protein folding
Source: PLoS One. 2021 Jan 13;16(1):e0244547. doi: 10.1371/journal.pone.0244547 (PMC7806123; doi:10.1371/journal.pone.0244547)
Supplement: Attachment — Submitted filename: reply.pdf [file pone.0244547.s005.pdf]

ref: PONE-D-20-18433

On topology and knotty entanglement in protein folding

Answers to Reviewers reports:

We quote the *Reviewers comments verbatim in red italics*, and after each comment we provide answers in black Roman type. Page and line numbers refer to the revised version of the manuscript.

We thank Reviewers for comments and proposals, that have helped us to improve our manuscript. We hope that the enclosed version answers all the points that were raised.

Reviewer 1:

The description of the multi-soliton approach should be more explanatory, then it would be more beneficial for the broader audience of researchers studying protein folding. For example, in equations (6) and (7), for an i-th C-alpha site: do parameters a, b, c, d, m, and lambda relate to the single soliton stretching over a given region to which the i-th site belongs? If yes, maybe these parameters should be indexed to underline their dependence on the corresponding soliton. In this form, these parameters seem to be constant for the whole chain.

We have fully rewritten the description of the multi-soliton approach, both in the article and in supplementary material. Please see the new, more explicit version of equation (5) and the two pages that follow; lines 120-179.

In my opinion the following fragment from Introduction (page 2/15) is misleading: ?In our approach an individual topological soliton profile describes a single super-secondary protein structure such as a helix-loop-helix or a strand-loop-strand motif.? This suggest that a single soliton stretches along a long fragment of the chain, while in this study, some solitons stretch over only 4 residues (based on data from Table I). The cited fragment probably refers to the ideal situation, but in this particular case it looks very different.

We have added a clarification - see text below the definition of β-strand, after equation (7). See also the re-written section in Supplementary Material. In particular *The number of individual soliton profiles that are introduced, depend on the desired precision of the multi-soliton: An increase in the number of individual solitons increases the number of parameters, and improves the precision.* Then, in the case of 2J6B, we have given a detailed explanation on the length/number of solitons, in the first paragraph of Results, lines 238-257. This is further elaborated on, in the Supplementary Material.

In short, we could decrease the number of solitons but then the RMSD between 2J6B and the multi-soliton might exceed the resolution at which the 2J6B has been measured. For us, to have a multi-soliton at RMSD distance comparable, or slightly below the resolution of the experimental structure is a good "rule-of-thumb" that we have a good multi-soliton. The better the accuracy, the less there are unknotted/misfolded final states.

Another good criterion for accuracy not used here but in some other articles, would be to compare the RMSD distance to Debye-Waller fluctuation distances, that can be computed from B-factors. The outcome would not be much different.

Note that 2J6B has a pretty complex structure, in comparison to many other globular proteins we have studied, such as myoglobin.

I suggest also to add a figure with the structure of the studied protein, with marked at least several fragments of the polypeptide chain related to the corresponding solitons (maybe even all solitons, since there are 20 solitons in total, according to the Table I in supplementary material) ? maybe as an additional panel in Figure 2.

Done: See Panel 2 c)

In Table I in supplementary material: what are the parameters lambda$_1$ and lambda$_2$, since everywhere else lambda appears without index (the same with parameters m$_1$ and m$_2$)?

Full explanation is added in Supplementary Material.

One simulation cycle consists of heating cooling cycle. Authors for the presented data performed 1000 of such cycles. However, this information is provided only in the caption of Figure 3. This should be moved to Methods or, the third paragraph of Results.

Done - see lines 220-226 in Methods.

The beta-bridge together with residue 61 should be marked in Figure 6 explicitly. Also fragments 74-80 and 93-99 should be indicated in the structure of the studied protein; otherwise, it si difficult to follow the described events in page 9/15.

Done

Minor remarks: (1) page 3/15, line 101: There is: ?From (1), (2)? - Did Authors mean equations (1), (3), and (4)? (2) page 5/15, line 176: ?Heating? ? ?heating? (3) page 6/15, line 205: Should be ?Figure 6 b)?. (4) page 9/15, line 239: ?Eve though? ? ?Even though?. (5) page 9/15, line 246, ??(4)? ? ?(Figure 4)?. (6) page 10/15, lines 273, 277: Authors refer to ?Figure 5

All done – thank you!

Reviewer 2:

1. Clearly the described approach is quite complicated, i.e. starting from NLS and working toward a multi-soliton solution.

We do not think this is very complicated. NLS equation is a widely studied, fundamental concept in soliton theory/integrability. There are many well known and developed techniques described in numerous textbooks, to analyze its multi-solitons, both in continuum and discrete.

Also, in Point 2. the Reviewer states him/herself that *"... the authors are making a very simple representation of a protein and then highlighting how simple the dynamics becomes."*

It is hard to see the advantages such an approach has over, for example, a much simpler method:

Reviewer writes a few lines later that *"... There is an additional simplification the authors make relative to Go-like approaches "*:

... start from a simple Go-like backbone, e.g. harmonic bond angles and cosine-like dihedrals and keep all frozen at the native values except for the 20 interface bond angles/dihedrals. Let these 20 interface angles vary, sampled however one likes, MD or MC. Maintain excluded volume however you want, but use e.g. the typical Go model approach. Ignore all native contacts. Unless I am missing something, I feel that this simplification of a Go model is nearly equivalent to your presented model. Could the authors please comment?

According to Sulkowska et.al. (our Reference [6]) it is not easy at all, to describe how 2J6B folds into a slipknot, even if one uses a much more detailed Go-model. We look forward that someone publishes how the model proposed by the Reviewer really works, in the case of a knotted protein. That would, for sure, be of interest to us and others, working on the subject.

Besides, in our approach the bond and torsion angles are not based on linear equations, they are not harmonic/cosine like. There are no solitons in any linear models. Solitons are products of nonlinearities, and our energy function has several nonlinear terms and these are essential to produce solitons: Our energy function is a variant of a textbook nonlinear soliton-supporting equation.

Note, in this case, there seems to be no advantage to the authors approach in terms of computational efficiency.

As a code/functioning model along the lines proposed by the Reviewer is not currently available, so we are not able to make a judgement.

2. The authors are motivated to fold a non-trivial protein fold in a ?computationally highly effective? way. In their quest they reduce a 109 residue protein into a chain with effectively 20 flexible pieces and 40 degrees of freedom. Certainly, this allows a much quicker sampling of the configuration space. I wonder though what the authors hope to learn from this.

We hope to learn the same what other authors do, when simulating the folding of knotted proteins: At the moment, a full all-atom molecular dynamics is not able to describe folding of knotted proteins,

such as the formation of slipknotted 2J6B. For this reason it remains a quest in the community to develop practical/pragmatic models to describe protein folding. In this regards, our hopes are no different than the hopes of all those who develop and apply coarse grained models. In the case of proteins with a knot, thus far only Go-models have been available. These models are even more coarse grained than ours, still we can – and have – learn a lot from the Go-model simulations.

The general question about knots in the protein folding field is how the proteins are able to fold a specific knot and avoid wrong topologies. Perhaps the authors wish to hypothesize that 2J6B has effectively only 40 degrees of freedom, and that is why such 2J6B can fold quickly. And that if a protein is modeled as having ¿200 degrees of freedom (e.g. just a Go-model backbone), then traps appear that are unphysical. In this case the authors would need to show how the choice of the number of rigid segments influences the dynamics and whether their representation maintains the essential features of a protein.

We do not think, nor claim that traps in Go-model are unphysical. We need to understand these traps, maybe they are biologically important. For that it should help to study the folding using different kind of models, like ours. And to compare the results , to eventually understand what is physical and what is not.

There is an additional simplification the authors make relative to Go-like approaches, they removed any frustration from long-sequence separated native contacts. These contacts are usually the ones causing issues in Go-like models (e.g. in Sulkowska 2009). Is there any theoretical justification for this?

Maybe there is a little misunderstanding here, due to our earlier sloppy description of the model that was also pointed out by Reviewer 1. We have now described the model in much more detail:

No, we don't remove such contacts. These are accounted for both by the last term in free energy (5), and by the non-local, extended character of a soliton. Please see the lines 137-143 and the lines 150-156 that we have added, to account for these comments. Our free energy is simply a systematic expansion of a generic full free energy, in terms of the distances between the interaction centers.

As it is, it appears the authors are making a very simple representation of a protein and then highlighting how simple the dynamics becomes.

On the one hand, the Reviewer states in point 1. that our approach is *complicated*, and on the other hand, he/she now says that it is *simple*.

The absence of individual atomic degrees of freedom is a reasonable approximation, made in many coarse grained approaches. At the same time, the soliton degrees of freedom effectively integrate the dynamics of individual atoms, in our approach.

3. The authors investigate folding/unfolding by progressively changing the temperature - essentially an annealing process. Using annealing to investigate kinetic pathways has certainly been used in the past, but typically as a crutch when the employed models are too slow to allow proper constant temperature sampling. I suppose that is not the case here. In order to make comments on kinetic aspects of folding, the temperature must be held constant.

We agree on thermalization issue, and that is why we use Glauber algorithm. It approaches a Gibbs state at exponential rate; in our simulation the chain thermalizes *very* quickly. Please see our ref. [23] for a detailed comparison of thermalization speed using different algorithms, in the present model.

We have added a clarification, in lines 180-183: We have in our mind folding and unfolding experiments, where protein structure is unfolded using a high temperature, and then observed how it folds when temperature is lowered to physiologically relevant values. Temperature is widely used as a variable in laboratory experiments on protein folding, as it is very difficult to make experiments by pulling out an unfolded protein from ribosome, in vivo. While we agree that a very high temperature is not biologically relevant it is a very effective way to prepare a protein in a non-native, unfolded conformation under laboratory conditions.

The anecdotes from Go-model simulations discussed the text come from studies investigating the topological barrier crossing at constant temperature.

Thank you for the clarification: We have added the text in lines 180-183 to make this clear.

4. In MC dynamics, there is no requirement that the sequence of sampled structures are kinetically related. Rather the desire is typically to find energetic minima. For example, while the potential doesn't allow chains to lie on top of each other, what prevents a single MC move from undergoing a chain crossing? The authors should comment on why their MC dynamics is indicative of a physically-realizable set of moves.

This is a very good point. Obviously, we have taken great care to handle this issue - mostly in our earlier articles on algorithm development: In our simulations, the probability of chain crossing is extremely small, we have never observed it. This is due to the large change in Glauber algorithm probability, for acceptance of large changes in angles: The Pauli repulsion has a range of 3.8 Å, and thus a chain crossing makes a big difference between residue positions, between consecutive steps - it would move a large number of residues by at least 7.6 Å. This would not be accepted by the Glauber algorithm, unless the two very different conformations happen to be essentially degenerate in energy.

A chain crossing be eliminated easily, by simply demanding that steps where coordinates of any Cα atom change by more than a certain amount, is not accepted. The algorithm as we implement it, makes the backbone motion to proceed more or less in a "continuous" fashion which, together with the Pauli exclusion, is enough to prevent chain crossing: No residue moves 3.8 Å, or more, at a given MC step.

5. There is no discussion of how the topology of the protein is determined. This needs to be explained.

We have added discussion in lines 220-226: We identify a knot by measuring the RMS distance between the final simulated structure and the crystallographic 2J6B. For this we compute the RMSD for the entire chain, and for the critical knotted region. We also make measurement of distances between key atoms. At the end, we have made extensive screenings visually, by hand, and we do this whenever there is any doubt: Admittedly this is not a very fast way, and not the smartest one. But at the same time a visual inspection is a fool-proof method: A full series of runs with 1.000 knots takes a few hours to check, visually.

We are aware that sites such as `Knotprot` use algorithms for knot detection and identification, and in future we plan to implement such techniques.

Also, some time traces of the topology versus time are necessary to get a feeling for the dynamics. For example, watching the movie the N-terminus appears to thread/unthread multiple times.

It is true, that the dynamics does not proceed smoothly in the movies. However, the fluctuations are physical, they are *nothing* but thermal fluctuations; the proteins are at final temperature, and this causes conformational fluctuations that we describe by the Glauber algorithm. The thermal movements also reflect ruggedness in the energy landscape. Time tracing beyond what it takes *in silico* would be difficult, for this we would need experimental data to set the scale. Also, we would need a reliable relation between the temperature factor β of the simulation, and physiological temperature factor kT that we do not have: We are sorry, but for time evolution the best we can do is provide the movie, that shows *verbatim* how the structure evolves with respect to *in silico* time.

The authors claim that the trefoil knot precedes the slipknot formation. Based on looking at the structure, this seems likely, but it needs to be quantified.

The quantification appears in text of Figure 5, that we have now improved: We have visually inspected our simulations, and the intermediate trefoil is consistently observed. Our statement about trefoil is observational one, and the movie we have added in Supplementary Material describes a generic trajectory. The peak in Figure 5 amounts to a numerical quantification of the presence of trefoils. It shows our data, with average and one-sigma deviation: All structures within one-sigma deviation from the average, at the peak, are trefoils.

A very precise (e.g. topological) quantification what is trefoil, is little problematic because strictly speaking, an open chain does not form a knot. But we trust that anyone looking at Figure 5 b) would agree, it is as much a trefoil as a protein backbone can be.

6. What do the trapped states in Fig6 B look like? I guess a couple folding trajectory movies

could be useful.

We have added two movies as an example of a trajectory that does not fold into slipknot. In one movie there is no knotted structure in the final conformation; this is the generic situation. In the other movie we show a misfolded state, where the protein gets trapped in the trefoil intermediate; these are extremely rare final structures in our simulations. See also text in Figure 6.

7. fig6 panel A, how is $\Delta\phi$ defined?

We have added clarification in the figure text.

Also how is the relationship degree C $<->$ log β determined?

Please see text in lines 267-272: We take the correspondence from Ref. [43]. For more details, we have added text in lines 272-275; in [42] it is explained in detail, how to compute β as a function of kT. Since this is not very simple (involves analysis of a renormalization group) we have not provided details as referens to original articles should suffice.

8. Why does the transition vary over 3 orders of magnitude in β? Taking units where kT=1 at the transition temperature, the width of the transition is typically no more than 10%, i.e. all folded to all unfolded between 0.9 and 1.1, same should be true for β.

The relation is not linear but entails an exponential; we have added reference to [42] in lines 272-275 where the relation between the two is derived in detail.

9. Question 4 might be related. Why are the values of d,a, etc so small in SI Table 1? It is suggested d$\sim$2 earlier. Perhaps a different set of units would make more sense?

We would have value $d \sim 2$ **IF** we would have standard textbook nonlinear Schrödinger equation, but that is not the case. We agree there is a big difference, but this is what comes out from our calculations. Partly it reflects the fact that in real proteins the bond angles are known to be very rigid, and torsion angles are highly flexible.

We agree that a different set of units could make the numbers look nicer - we need to take this discussion up with the developers of the software; maybe an adjustment how the Glauber algorithm computes probabilities would do. We have added a link to the software in lines 233-235.

10. Fig 4 would be a quite surprising result based on previous publications that show threading is a quite slow and frustrated process. Of course, in the present model there is no frustration at all, so the protein is free to deeply thread. However, from a kinetic point of view, it is strange that the protein would first deepen the threading of the C-terminal portion before unthreading it.

This is what comes out from our simulations. Certainly, there is work to be done, also to understand differences between different approaches. This is how progress will be made.

Just a comment: I suspect that for more deeply knotted proteins, e.g. YibK, the authors will have similar difficulties in folding compared to Go-like approaches - likely even more problems.

We are working on more deeply knotted proteins, including YibK. Thus far we have not encountered more problems or serious difficulties, but the work is still in progress.

2J6B has very shallow threading of the N-terminus, which is what creates the non-trivial topology. It seems that deeper knots will require distortions of the backbone (e.g. temporary slipknots), which the rigid treatment here will prevent.

This is not clear to us, a priori. We need to wait and hope to see what future studies reveal.
